# Fabrication and Histological Evaluation of Porous Carbonate Apatite Block from Gypsum Block Containing Spherical Phenol Resin as a Porogen

**DOI:** 10.3390/ma12233997

**Published:** 2019-12-02

**Authors:** Yuta Sakemi, Koichiro Hayashi, Akira Tsuchiya, Yasuharu Nakashima, Kunio Ishikawa

**Affiliations:** 1 Department of Orthopedic Surgery, Graduate School of Medical Sciences, Kyushu University, 3-1-1 Maidashi, Higashi-ku, Fukuoka 812-8582, Japan; yasunaka@ortho.med.kyushu-u.ac.jp; 2 Department of Biomaterials, Faculty of Dental Science, Kyushu University, 3-1-1 Maidashi, Higashi-ku, Fukuoka 812-8582, Japan; khayashi@dent.kyushu-u.ac.jp (K.H.); tsuchiya@dent.kyushu-u.ac.jp (A.T.); ishikawa@dent.kyushu-u.ac.jp (K.I.)

**Keywords:** carbonate apatite, porous structure, bone regeneration, bone graft, osteoconduction

## Abstract

The utility of carbonate apatite (CO_3_Ap) as a bone substitute has been demonstrated. The feasibility of fabricating macroporous CO_3_Ap was evaluated through a two-step dissolution–precipitation reaction using gypsum as the precursor and spherical phenol resin as the porogen. Porogen-containing gypsum was heated to burn out the porogen and to fabricate macroporous structures. Gypsum transformed into CaCO_3_ upon immersion in a sodium carbonate solution, while maintaining its macroporous structure. Next, CaCO_3_ transformed into CO_3_Ap upon immersion in a Na_2_HPO_4_ solution while maintaining its macroporous structure. The utility of the macroporous CO_3_Ap for histologically reconstructing bone defects was evaluated in rabbit femurs. After 4 weeks, a much larger bone was formed inside the macroporous CO_3_Ap than that inside non-macroporous CO_3_Ap and macroporous hydroxyapatite (HAp). A larger amount of bone was observed inside non-macroporous CO_3_Ap than inside macroporous HAp. The bone defects were completely reconstructed within 12 weeks using macroporous CO_3_Ap. In conclusion, macroporous CO_3_Ap has good potential as an ideal bone substitute.

## 1. Introduction

In orthopedic surgery, bone grafts have been used for the treatment of pseudarthrosis or bone defects due to bone fracture or tumor resection. Autograft is still the gold standard. However, although autografts possess good osteoconduction and osteoinduction and induce osteogenesis without causing an immunological response, more skin incision is required and bone harvest is limited [1,2,3,4]. Therefore, artificial bone substitutes are often used to fill bone defects in combination with or without autografts.

Hydroxyapatite (Hap; Ca_10_(PO_4_)_6_(OH)_2_) has been widely used as an artificial bone substitute because it exhibits high tissue compatibility and good osteoconduction. Contrary to autografts, HAp is hardly absorbed at the bone defect and remains there for a long time [5]. It should be noted that the inorganic component of the bone is not pure HAp but AB-type carbonate apatite (CO_3_Ap; Ca_10-a_(PO_4_)_6-b_(CO)_c_), which contains 4–9% carbonate ions (CO_3_^2−^) in an apatitic structure [6]. In addition, resorption is known to be quicker with an increase in substitution of CO_3_^2−^ at the A-site or OH site and/or at the B-site or PO_4_ site [7]. Furthermore, compared to the crystal structure of HAp, the structure of CO_3_Ap more closely resembles bone apatite [8]. Thus, CO_3_Ap is expected to be an ideal bone substitute.

Recently, CO_3_Ap blocks have been fabricated in aqueous solution through a dissolution–precipitation reaction using precursor blocks such as CaCO_3_ [9,10,11,12,13,14,15,16], dicalcium phosphate dihydrate (CaHPO_4_·2H_2_O) [17,18], α-Ca_3_(PO_4_)_2_ [19,20,21,22,23,24], and CaSO_4_ [25,26,27,28]. The fabrication of CO_3_Ap blocks occurs through a simple process. Upon immersion in an aqueous Na_2_HPO_4_ solution, the CaCO_3_ block dissolves and releases Ca^2+^ and CO_3_^2−^ into the aqueous solution. No further reaction will occur in the absence of other ions. However, when the solution contains phosphate, it becomes supersaturated with respect to CO_3_Ap. Therefore, Ca^2+^ and CO_3_^2−^ released from CaCO_3_ are precipitated as CO_3_Ap in the phosphate solution. As a consequence of this dissolution–precipitation reaction, the CaCO_3_ block becomes a CO_3_Ap block while maintaining its macroscopic structure. The fabricated CO_3_Ap block thus shows properties similar to the properties of the bone. In contrast to sintered HAp, which is not resorbed by osteoclasts, CO_3_Ap blocks are resorbed by osteoclasts and they can serve as a new bone replacement [9,29,30,31]. Furthermore, CO_3_Ap blocks were found to upregulate osteoblastic differentiation and they showed a much higher osteoconductivity than HAp [32].

The structural properties of bone substitutes including porosity, pore size, interconnectivity, and geometry are important factors that govern their bone regeneration ability [33,34]. Macropores with diameters greater than 100 µm ensure nutrient supply, cell colonization, and metabolic waste transport [35]. Porous CO_3_Ap blocks are expected to exhibit superior bone regeneration ability compared to current dense CO_3_Ap bone substitutes. Among various precursors for the fabrication of CO_3_Ap blocks, gypsum (CaSO_4_) is unique since it shows self-setting ability, which enables it to be shaped into any structure, and it maintains stability at high temperatures, which are required to burn out polymeric porogens.

The objective of this investigation was to evaluate the feasibility of fabricating porous CO_3_Ap blocks using gypsum and polymeric porogens and to explore their utility with respect to physical and histological properties.

## 2. Materials and Methods

### 2.1. Fabrication of Cylindrical CO_3_Ap and HAp Blocks

Cylindrical porous CO_3_Ap blocks (6 mm in diameter and 3 mm in thickness) were fabricated using calcium sulfate hemihydrate (CaSO_4_·1/2H_2_O, Wako Pure Chemical Industries Ltd., Osaka, Japan) and spherical phenol resin (LPS-C100; Lygnyte Inc., Osaka, Japan), with an average diameter of 100 µm by compositional transformation through dissolution–precipitation reactions, which converted CaSO_4_ to CaCO_3_ and CaCO_3_ to CO_3_Ap.

CaSO_4_·1/2H_2_O powder and spherical phenol resin were mixed so that the proportion of spherical phenol resin was 30% and 40% by mass. The mixture or CaSO_4_·1/2H_2_O powder alone was further mixed with distilled water at a water-to-powder ratio of 0.23. The paste thus prepared was packed into a split plastic mold (6 mm in diameter and 3 mm in height), and both sides were covered with glass plates and kept at room temperature for 3 h for hardening. The hardened sample was heated to 700 °C at a rate of 0.13 °C/min and maintained at 700 °C for 3 h to burn out the spherical phenol resin.

The CaSO_4_ blocks were then immersed in a solution of 2 mol/L sodium carbonate (Na_2_CO_3_) and 2 mol/L sodium hydrogen carbonate (NaHCO_3_) adjusted to pH 9 at 90 °C for 24 h to fabricate CaCO_3_ blocks through the dissolution–precipitation reaction using CaSO_4_ blocks as the precursor. The solution is denoted as Na–H–CO_3_ in the remainder of the text.

Next, CaCO_3_ blocks were immersed in 1 mol/L disodium hydrogen phosphate (Na_2_HPO_4_) at 80 °C for three weeks to fabricate CO_3_Ap blocks through the dissolution–precipitation reaction using CaCO_3_ blocks as a precursor.

Cylindrical porous HAp blocks (6 mm in diameter and 3 mm in height) were fabricated as a control. HAp powder (HAP-200; Taihei Chemicals, Saitama, Japan) was mixed with spherical phenol resin porogens so that the mass proportion of the porogens was 40%. The mixture was pressed at 20 MPa with a stainless-steel mold using an oil pressure press machine (MT-50HD; NPa System, Saitama, Japan). The compacts were heated at 0.13 °C/min to 1000 °C and maintained at 1000 °C for 3 h in an electronic furnace (SBV-1515D; Motoyama, Osaka, Japan) to burn out the porogens and to sinter the HAp powder. The mass content of spherical phenol resin in the original precursor is stated in parentheses. For example, CO_3_Ap (30%) indicates that the CO_3_Ap block was made using 30% spherical phenol resin by mass in the raw material, i.e., CaSO_4_·1/2H_2_O.

### 2.2. Characterization of Samples

The composition of the samples was analyzed by X-ray diffraction (XRD), Fourier transform infrared (FT-IR) spectroscopy, and elemental analysis. XRD patterns were recorded using a powder X-ray diffractometer (D8 Advance A25; Bruker AXS GmbH, Karlsruhe, Germany) with CuKα radiation operated at a tube voltage of 40 kV and a tube current of 40 mA during continuous scanning at 2θ, ranging from 10° to 40° at a scanning rate of 2°/min. FT-IR spectra were measured by a KBr disc method using a spectrometer (SPECTRUM 2000LX; Perkin Elmer Co. Ltd., Kanagawa, Japan). 

The surface morphologies of the CO_3_Ap and HAp blocks were observed using a scanning electron microscope (SEM) (S-3400N; Hitachi High-Technologies, Tokyo, Japan) at an accelerating voltage of 15 kV after applying a gold–palladium coating with a magnetron sputtering machine (MSP-1S; Vacuum Device Co., Ibaraki, Japan). 

The distribution of the interconnected pore size was evaluated through the penetration of Hg vapor using porosimetry.

The overall porosity was calculated by dividing the apparent density of the sample (d_samp_) by the theoretical density of HAp (d_HAp_), as shown below in Equation (1).

Porosity (%) = 100 − 100 (d_samp_/d_HAp_) (1)

The mechanical strength of the samples was evaluated in terms of diametral tensile strength (DTS). The diameter and height of each sample were measured using a micrometer (MDC-25M; Mitutoyo Co. Ltd., Kanagawa, Japan). A load was applied to crush each sample in a universal testing machine (AGS-J; Shimadzu Corp., Kyoto, Japan) at a crosshead speed of 1 mm/min. Each DTS value represents the mean of at least eight samples. 

### 2.3. Surgical Procedure

Sixteen 18-week-old white, male Japanese rabbits with an average weight of 3.1 kg (Japan SLC Inc., Hamamatsu, Japan) were subjected to animal studies. The experiments were conducted according to the Guide for the Care and Use of Laboratory Animals, National Research Council, USA; the protocols were approved by the Animal Care and Use Committee of Kyushu University (approval number A-28-144-1). The CO_3_Ap and HAp blocks were sterilized by heating at 170 °C for 3 h.

The rabbits were anesthetized by intramuscular injection of ketamine-xylazine (35–10 mg/kg). A medial longitudinal skin incision was made at the distal femur of both legs, and the medial epicondyle of the femur was exposed. A 5.8 mm hole was drilled into the epiphysis of the distal femur and extended to 6 mm. After the CO_3_Ap and HAp blocks were inserted, the periosteum, fascia, and skin were closed by suturing. All animals were allowed unrestrained movement after recovery from anesthesia.

### 2.4. Image Analysis

Four and twelve weeks after implantation, the rabbits were euthanized and the distal femurs were harvested (n = 4 in each group) while removing the surrounding soft tissue. The distal femur including the CO_3_Ap and HAp blocks were scanned using micro-computed tomography (μ-CT) (Skyscan 1075 KHS; Skyscan, Kontich, Belgium) at a source voltage of 60 kV and a source current of 170 μA with a 0.5 mm aluminum filter. Slices obtained from μ-CT measurements were used to reconstruct 2D and 3D images using analysis software. The volume of the remaining blocks was calculated by quantitative 3D evaluation. Before implantation, the volume of each block was calculated using its diameter and thickness. The residual rate (%) was calculated using Equation (2).

Residual rate (%) = (volume of remaining block/volume of block before implantation) × 100(2)

The trabecular thickness (Tb, Th) and trabecular number (Tb, N) were analyzed using software provided with the μ-CT scanner. The trabecular thickness and trabecular number were calculated using Equations (3) and (4), respectively.

Tb, Th = 2 × bone volume (BV)/bone surface (BS) (3)

Tb, N = BV/(total tissue volume × Tb, Th)(4)

### 2.5. Histological Procedures

The obtained samples were fixed with a solution containing 4% paraformaldehyde and 5% glutaraldehyde in 0.1 mol/L phosphate-buffered saline at pH 7.4 for one week. After decalcification with Plank–Rychlo (FUJIFILM Wako Pure Chemical Corporation, Osaka, Japan) solution for 24 h, the samples were dehydrated by a graded series of ethanol and N-butyl glycidyl ether followed by embedding in an epoxy resin (Quetol 651; Nisshin EM, Tokyo, Japan). The samples were sectioned at a thickness of 1 μm using an ultramicrotome (ULTRACUT S; Reichert-Nissei, Tokyo Japan), followed by staining with hematoxylin and eosin (H&E). Next, the specimens were examined under a microscope (BZ-X710; KEYENCE, Osaka, Japan) to detect grafted material and new bone formation. The amount of new bone was estimated as a percentage of the sample insertion area using a BZX analyzer attached to the microscope.

### 2.6. Statistical Analysis 

The Mann–Whitney U test was performed to assess the difference in the residual rate, new bone formation, trabecular thickness, and trabecular number. Significant difference was defined as *p* < 0.05. Statistical analyses were performed using the JMP software (Version 12.0; SAS Institute, Cary, NC, USA).

## 3. Results

**Figure 1:**
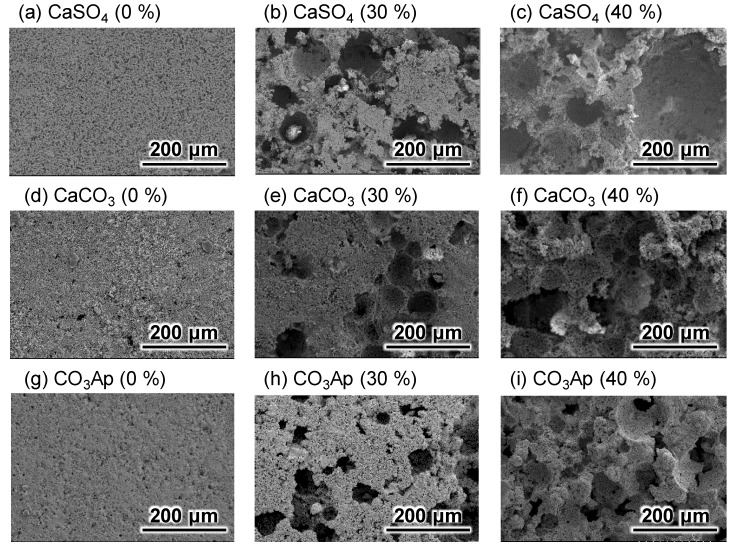
SEM images of gypsum (CaSO_4_) blocks (**a**–**c**), CaCO_3_ blocks (**d**–**f**), and carbonate apatite (CO_3_Ap) blocks (**g**–**i**).

Figure 1 shows the SEM images of CaSO_4_ blocks before (Figure 1a–c) and after immersion in Na–H–CO_3_ (Figure 1d–f) and Na_2_HPO_4_ solution (Figure 1g–i). The macrostructure of the samples was the same before and after immersion in Na–H–CO_3_ and Na_2_HPO_4_ solutions, whereas the microstructure was different. Needle-like crystals typical of CaSO_4_ were observed before immersion. When the CaSO_4_ blocks were immersed in Na–H–CO_3_ solution, rhombohedral and hexagonal crystals were observed. When the samples were further immersed in Na_2_HPO_4_ solution, fine aggregated crystals were observed.

**Figure 2:**
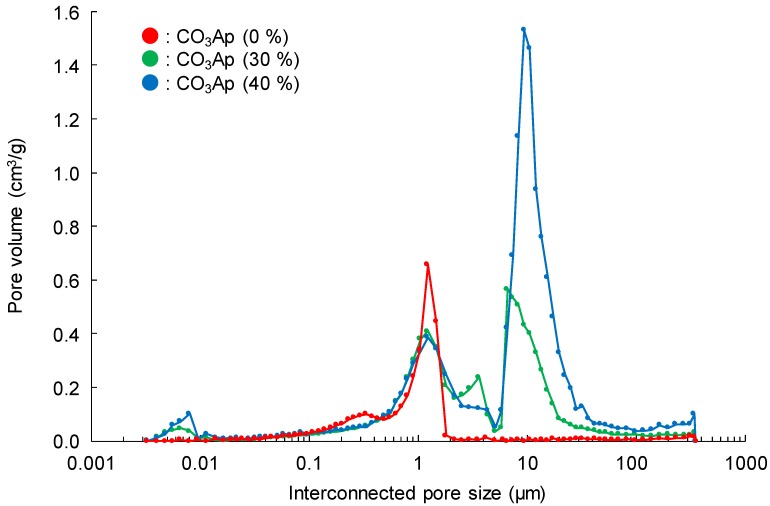
Pore size distribution of CO_3_Ap blocks measured by mercury intrusion porosimetry.

Figure 2 summarizes the pore size distribution measured by mercury intrusion porosimetry. In the case of CO_3_Ap (30%) and CO_3_Ap (40%), interconnected pores with diameters of 5–30 μm were fabricated in addition to pores that were 0.2–2 μm in diameter, which were observed also in CO_3_Ap (0%).

**Figure 3:**
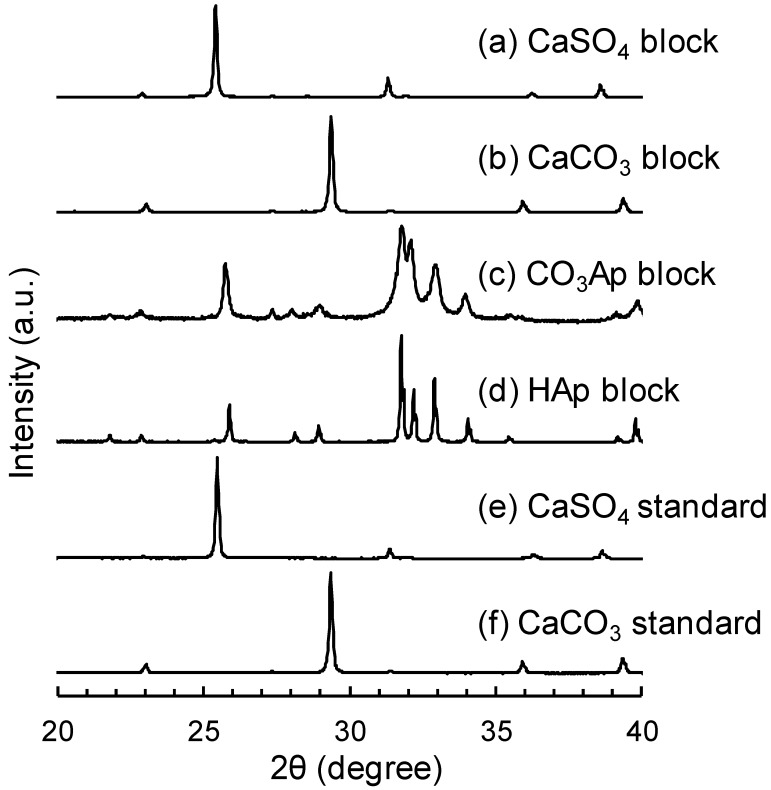
XRD patterns of (**a**) CaSO_4_ block, (**b**) CaCO_3_ block, (**c**) CO_3_Ap block, and (**d**) hydroxyapatite (HAp) block compared to XRD patterns of (**e**) CO_3_Ap powder and (**f**) CaCO_3_ block.

Figure 3 summarizes the XRD patterns of CaSO_4_ blocks before (Figure 3a) and after immersion in Na–H–CO_3_ (Figure 3b) and Na_2_HPO_4_ solution (Figure 3c) and those of sintered HAp blocks (Figure 3d). The XRD patterns of standard CaSO_4_ (Figure 3e) and CaCO_3_ (Figure 3f) are also presented for comparison. As shown in this Figure, CaCO_3_ blocks were fabricated through the dissolution–precipitation reaction when CaSO_4_ blocks were immersed in Na–H–CO_3_ solution. Next, apatite blocks were fabricated through the dissolution–precipitation reaction when CaCO_3_ blocks were immersed in Na_2_HPO_4_ solution (data not shown).

Apatite blocks fabricated in Na_2_HPO_4_ solution using CaCO_3_ blocks as a precursor exhibited broader peaks compared with the peaks for sintered HAp, indicating that the crystallinity of apatite formed in the Na_2_HPO_4_ solution was lower or closer to that of bone apatite. The XRD patterns were the same regardless of the amount of added spherical phenol resin.

**Figure 4:**
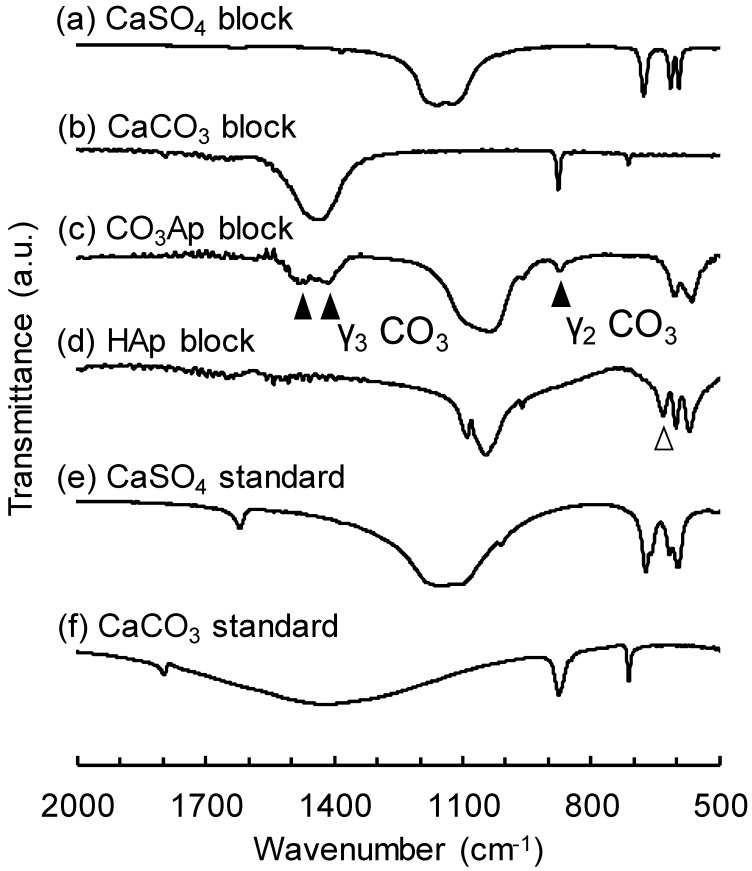
FT-IR spectra of (**a**) CaSO_4_ block, (**b**) CaCO_3_ block, (**c**) CO_3_Ap block, and (**d**) HAp block compared to X-ray diffraction (XRD) patterns of (**e**) CO_3_Ap powder and (**f**) CaCO_3_.

Figure 4 summarizes the FT-IR spectra of CaSO_4_ blocks before (Figure 4a) and after immersion in Na–H–CO_3_ (Figure 4b) and Na_2_HPO_4_ solution (Figure 4c) and those of sintered HAp blocks (Figure 4d). The FT-IR spectra of standard CaSO_4_ (Figure 4e) and CaCO_3_ (Figure 4f) are also presented for comparison. When CaCO_3_ blocks were immersed in Na_2_HPO_4_ solution, the appearance of CO_3_ peaks that were attributed to AB-type CO_3_Ap (▲) [36,37] indicated that AB-type CO_3_Ap was formed. No absorption peak due to apatitic OH was found. In contrast, sintered HAp exhibited absorption peaks attributed to OH (△), but no peaks attributed to apatitic CO_3_ were observed.

The porosity and DTS values of CaSO_4_, CaCO_3_, CO_3_Ap, and HAp blocks are summarized in Table 1. The porosity of these blocks decreased with increase in the amount of spherical phenol resin added. The porosity of CaCO_3_ blocks was higher compared to those of CaSO_4_ and CO_3_Ap blocks regardless of the amount of spherical phenol resin added because of the presence of micropores in addition to macropores. The DTS value decreased with increasing porosity. The DTS value of the CaCO_3_ blocks was lower than that of the CaSO_4_ blocks, while that of the CO_3_Ap blocks was higher than that of the CaCO_3_ blocks.

**Figure 5:**
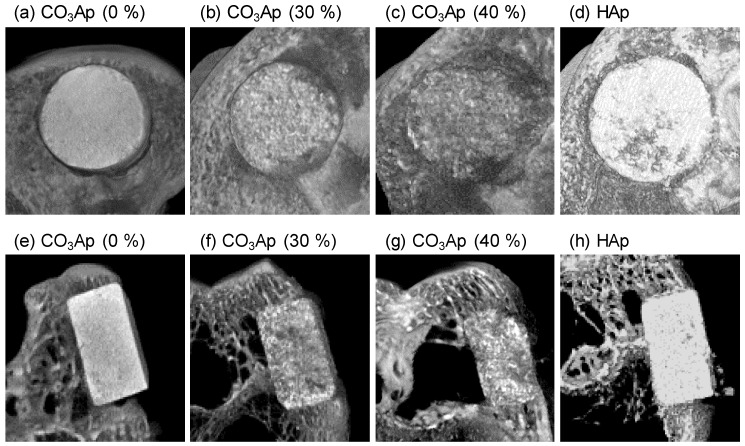
Micro-computed tomography (μ-CT) images of (**a**−**c**,**e**–**g**) CO_3_Ap blocks and (**d**,**h**) HAp blocks at 4 weeks after implantation in the (**a**–**d**) sagittal plane and (**e**–**h**) coronal plane of the rabbits’ distal femurs.

**Figure 6:**
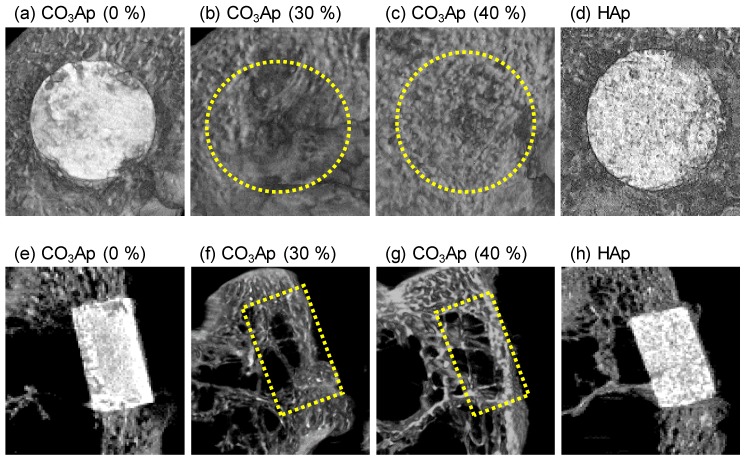
μ-CT images of (**a**−**c**,**e**–**g**) CO_3_Ap blocks and (**d**,**h**) HAp blocks at 12 weeks after implantation in the (**a**–**d**) sagittal plane and (**e**–**h**) coronal plane of the rabbits’ distal femurs: Yellow dotted lines indicate the implant insertion portions.

Figure 5 and Figure 6 summarize the μ-CT observations for CO_3_Ap (5a–c, 5e–g, 6a–c, 6e–g) and HAp blocks (5d, 5h, 6d, 6h) in the sagittal (5a–d, 6a–d) and coronal planes (5e–h, 6e–h) at 4 and 12 weeks, respectively. The edges of CO_3_Ap (30%) and CO_3_Ap (40%) were rounded at 4 weeks after implantation, whereas the CO_3_Ap (0%) and HAp (40%) blocks had a rectangular shape even at 12 weeks in the coronal plane. At 12 weeks, both CO_3_Ap (30%) and CO_3_Ap (40%) were resorbed almost completely and replaced with new bone. Spotty high-density regions were observed in CO_3_Ap (30%) and CO_3_Ap (40%) at 4 weeks and in CO_3_Ap (0%) at 12 weeks, particularly on the cancellous bone side. This suggested that osteointegration occurred earlier in CO_3_Ap (30%) and CO_3_Ap (40%) compared to that in the CO_3_Ap (0%) and HAp blocks (40%). Additionally, a greater amount of spotty high-density regions was found on the cancellous bone side than at the cortex. In contrast, spotty low-density regions were formed in the HAp blocks at 12 weeks after implantation. 

**Figure 7:**
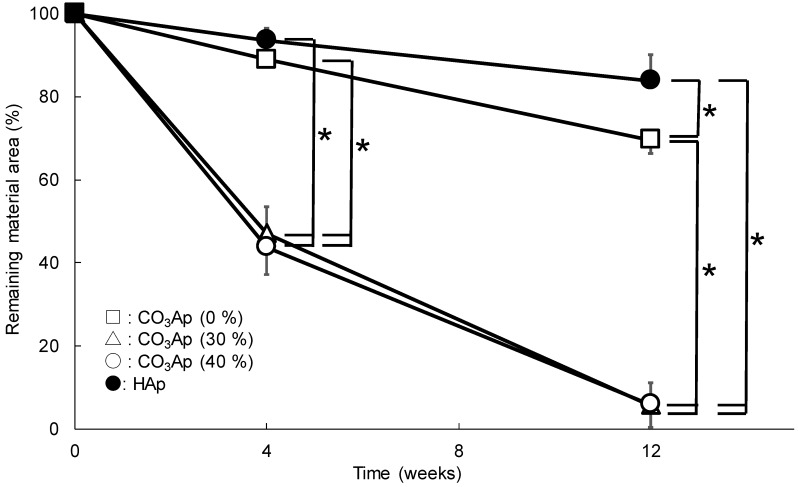
Remaining material area (%) in the defect at 4, 8, and 12 weeks after implantation of CO_3_Ap and HAp blocks (*p < 0.05).

Figure 7 shows the amount of residual materials in the CO_3_Ap and HAp blocks. The amounts of residual CO_3_Ap (30%) and CO_3_Ap (40%) were statistically lower (*p* < 0.05) than those of CO_3_Ap (0%) and HAp (40%). In addition, the amount of remaining CO_3_Ap (0%) was statistically lower (*p* < 0.05) than that of HAp (40%). CO_3_Ap (30%) and CO_3_Ap (40%) blocks were almost completely resorbed at 12 weeks after implantation. 

**Figure 8:**
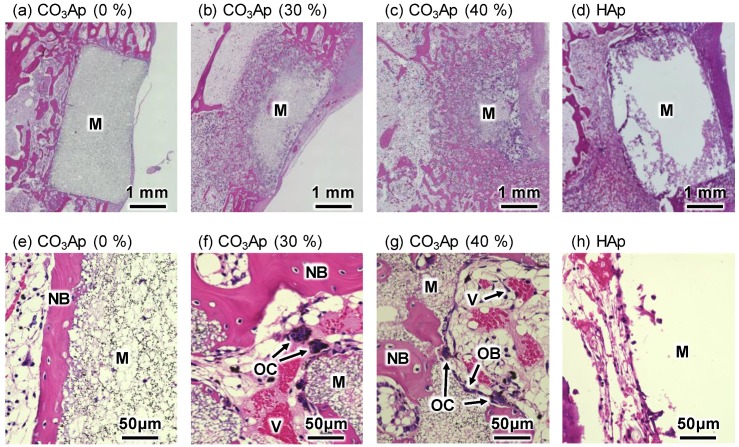
Hematoxylin and eosin (H&E)-stained histological images of the distal femurs with (**a**,**e**) CO_3_Ap (0%), (**b**,**f**) CO_3_Ap (30%), (**c**,**g**) CO_3_Ap (40%), and (**d**,**h**) HAp blocks at 4 weeks after implantation. M: material; NB: new bone; OB: osteoblast; OC: osteoclast; V: vessel-like tissue.

**Figure 9:**
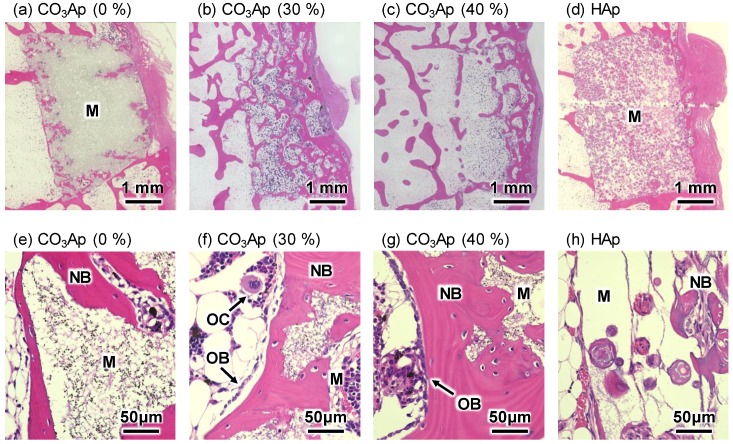
H&E stained histological images of the distal femurs including (**a**,**e**) CO_3_Ap blocks (0%), (**b**,**f**) (30%), (**c**,**g**) (40%), and (**d**,**h**) HAp blocks at 12 weeks after implantation. M: material, NB; new bone, OB; osteoblast, OC; osteoclast.

Figure 8 and Figure 9 summarize the H&E-stained histological images of the distal femurs 4 and 12 weeks after implantation. At 4 weeks, new bone was found on the surface of all CO_3_Ap samples, demonstrating the good osteoconductivity of CO_3_Ap. A small amount of new bone was also found on the surface of HAp (40%), indicating that HAp also exhibited osteoconductivity, even though the degree of osteoconductivity was limited for HAp compared with that for CO_3_Ap.

For macroporous CO_3_Ap or CO_3_Ap (30%) and CO_3_Ap (40%), tissues including new bone were formed inside the CO_3_Ap block. A much greater amount of tissues and cells including new bone, vessels, and red blood cells were found inside the CO_3_Ap (40%) block compared to those found in the CO_3_Ap (30%) block. In the case of CO_3_Ap (0%), no tissue penetration was observed at this stage. In the case of HAp (40%), fibrous tissue penetration was observed, whereas no bone tissue was seen inside the block.

Osteoclasts were also found inside the CO_3_Ap (30%) and CO_3_Ap (40%) blocks, and resorption occurred well from the trabecular bone side.

At 12 weeks, almost all the CO_3_Ap (30%) and CO_3_Ap (40%) blocks were resorbed. Moreover, the original trabecular structure was reconstructed, indicating that remodeling was completed within 12 weeks. New bone was also found inside the CO_3_Ap (0%) and HAp (40%) blocks. As can be seen in Figure 9h, new bone was spherical and stained more strongly by hematoxylin in the case of HAp (40%).

**Figure 10:**
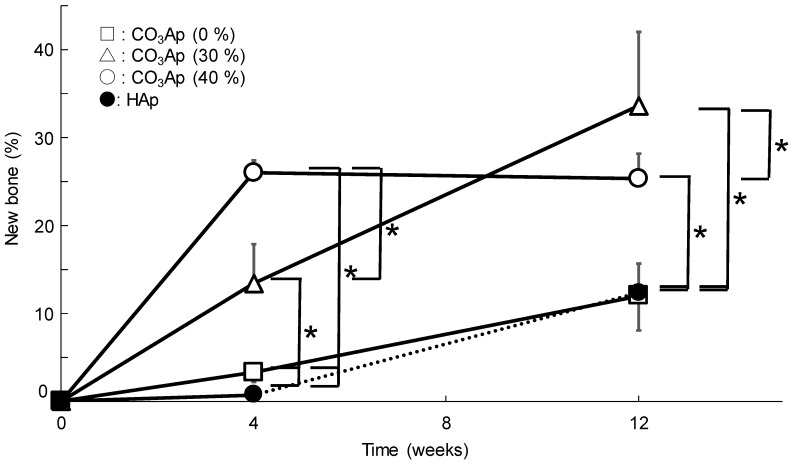
New bone area (%) in the defect at 4, 8, and 12 weeks after implantation of CO_3_Ap and HAp blocks (*p < 0.05).

Figure 10 illustrates the amount of new bone as a function of implantation period. The largest bone was formed in the case of CO_3_Ap (40%), followed by CO_3_Ap (30%) at 4 weeks. At 12 weeks after implantation, the amount of new bone inside the sample was increased compared with that at 4 weeks, except for that in CO_3_Ap (40%).

**Figure 11:**
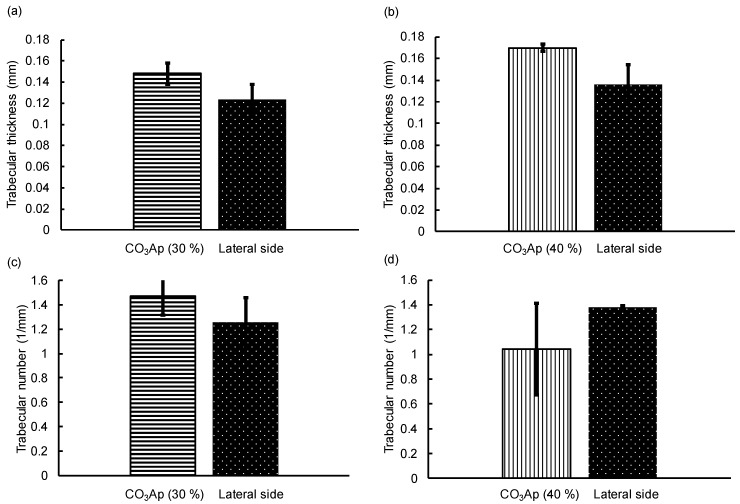
(**a**,**b**) Trabecular thickness and (**c**,**d**) trabecular number of the inserted portions of (**a**,**c**) CO_3_Ap (30%) and (**b**,**d**) CO_3_Ap (40%) blocks and the lateral side of the distal femurs.

Figure 11 shows the trabecular thickness (Tb, Th) and trabecular number (Tb, N) calculated in the implant insertion portions at 12 weeks to estimate the remodeling of new bone. At the trabecular bone of the femur epicondyle, femur Tb, Th and Tb, N were calculated as a control (Figure 8). There was no significant difference between Tb, Th and Tb, N in the implant insertion portion and that in the lateral side. This indicated that remodeling was almost complete within 12 weeks in the cases of CO_3_Ap (30%) and CO_3_Ap (40%) blocks.

## 4. Discussion

The results obtained in this study clearly demonstrated that porous CaSO_4_ blocks can be fabricated by heating the set CaSO_4_·2H_2_O containing spherical porogens since CaSO_4_ blocks are stable even at 700 °C. Porous CO_3_Ap blocks were fabricated through dissolution–precipitation reactions using CaSO_4_ blocks while maintaining the latter’s macroscopic structure. First, the CaSO_4_ block transformed into CaCO_3_ while maintaining its macroscopic structure when immersed in Na–H–CO_3_ solution (Figure 1, Figure 3 and Figure 4). Next, the CaCO_3_ block transformed into CO_3_Ap while maintaining its macroscopic structure when immersed in Na_2_HPO_4_ solution. The CO_3_Ap fabricated in this study was AB-type CO_3_Ap or CO_3_Ap found in the human bone. Notably, compared to HAp, this CO_3_Ap is more similar to bone apatite not only with respect to composition but also with respect to crystallinity. Both types of CO_3_Ap, i.e., bone apatite and CO_3_Ap, fabricated in this study are formed in an aqueous solution, whereas HAp is fabricated in an electronic furnace by sintering at high temperature. Therefore, the crystallinity of CO_3_Ap is close to that of bone apatite whereas that of HAp is extremely high, as shown in Figure 3. The dissolution–precipitation procedure is simple and enables the fabrication of artificial bone substitutes that are similar to bone in terms of composition and crystallinity.

The porosity and DTS were controllable by adjusting the amount of spherical phenol resin added. The DTS values of CO_3_Ap (0%), CO_3_Ap (30%), and the HAp block were 3.5 ± 0.3 MPa, 1.4 ± 0.2 MPa, and 1.8 ± 0.3 MPa, respectively. Kopperdahl and Keaveny reported that the DTS of the human trabecular bone lies between 1.3–3.5 MPa [38]. Thus, the DTS values of CO_3_Ap (0%), CO_3_Ap (30%), and the HAp block are nearly equal to that of human trabecular bone.

In addition, the histological results obtained in this study demonstrated the utility of microporous structures. Although CO_3_Ap (0%) was gradually replaced by new bone (Figure 10), the process took time. As shown in Figure 2, only micropores smaller than 2 μm were observed in the case of CO_3_Ap (0%). These micropores may be useful in facilitating osteoclastic resorption by decreasing the density of CO_3_Ap. However, they are too small for cells to penetrate the interior of the sample. Thus, osteoclasts need to resorb CO_3_Ap from the surface of the block, followed by new bone formation by osteoblasts. In contrast, macropores that are 5–100 μm in diameter are present in addition to micropores (smaller than 2 μm) in CO_3_Ap (30%) and CO_3_Ap (40%) blocks. The macropores allow cells and tissues to penetrate the interior of the sample. Although both CO_3_Ap (30%) and CO_3_Ap (40%) have macropores, more tissues penetrated CO_3_Ap (40%). As shown in Figure 8, tissues penetrated from the trabecular bone but not from the periosteum side. Due to cell and tissue penetration, replacement of CO_3_Ap by new bone, similar to remodeling, was remarkable for CO_3_Ap (30%) and CO_3_Ap (40%). At 4 weeks, the remaining CO_3_Ap (30%) and CO_3_Ap (40%) was approximately one fifth of that of CO_3_Ap (0%). The amount of new bone was much greater in CO_3_Ap (40%) compared to that in CO_3_Ap (30%), as shown in Figure 10. Additionally, the amount of new bone was much greater in CO_3_Ap (30%) compared to that in CO_3_Ap (0%). It should be noted that the amount of new bone formed in CO_3_Ap (0%) was greater compared to that in HAp (40%). This difference confirmed that the osteoconductivity of CO_3_Ap was much higher than that of HAp. Although the detailed mechanism of the higher osteoconductivity of CO_3_Ap has not yet been elucidated, one of the reasons behind this phenomenon could be the upregulation of differentiation from bone marrow to osteoblasts in the case of CO_3_Ap [32].

At 12 weeks after implantation, CO_3_Ap (30%) and CO_3_Ap (40%) were completely resorbed, as shown in Figure 7 and Figure 9. If CO_3_Ap (30%) and CO_3_Ap (40%) were resorbed linearly with time, similar to the resorption of CO_3_Ap (0%) and HAp (40%), complete resorption may have been achieved at approximately 8 weeks. This resorption rate seems sufficient for clinical purposes. Approximately 30% of CO_3_Ap (0%) was resorbed at 12 weeks. Although this value is much smaller than that for CO_3_Ap (30%) and CO_3_Ap (40%), the rate is twice that for HAp (40%) and acceptable for clinical applications. As a result of complete resorption and new bone formation, the bone defect area was reconstructed completely. Figure 11 summarizes the comparison of trabecular thickness and trabecular number between the reconstructed side and the lateral side used as a control. There was no statistically significant difference between them, indicating the complete reconstruction of the bone defect using CO_3_Ap (30%) and CO_3_Ap (40%).

In this study, spherical phenol resin with a diameter of 100 μm was used as a porogen. No optimization of the macropore size was carried out even though osteoconduction is known to be governed by pore size. Several researchers suggested that 0.3–0.5 mm is the optimal size range for osteoconduction [39,40]. At the same time, some studies proposed that pore sizes greater than 0.4 mm are less conducive to new bone formation, as evidenced by the accumulation of adipocytes and bone marrow [41] and reduced mechanical properties [39]. In general, 80–100 µm is the minimal pore size required for osteoconduction [42,43,44]. Further studies are needed to optimize the 3D interconnected porous CO_3_Ap based on the results of this investigation.

The total porosity of the HAp (40%) blocks fabricated was 64.2 ± 1.0%, which was close to that of the CO_3_Ap block (30%) at 67.5 ± 0.7%. The DTSs of HAp (40%) and CO_3_Ap (30%) were 1.8 ± 0.3 MPa and 1.4 ± 0.2 MPa, respectively, with no statistically significant difference. On the other hand, the resorption rate of CO_3_Ap (30%) was approximately eight times greater than that of HAp (40%) at 4 weeks and approximately six times greater at 12 weeks. This difference in resorption rate was probably due to the osteoclasts and not to chemical dissolution because, among calcium phosphates, apatite (including CO_3_Ap) is the most thermodynamically stable phase [9].

From the histological images, implant resorption, vascularization, and new bone formation were observed inside the CO_3_Ap (30%) blocks at 4 weeks and normal trabecular bone structure was seen at 12 weeks. Implant resorption occurred well from the trabecular side. This indicated that the resorption of the CO_3_Ap block was due to osteoclasts because, in the natural bone, remodeling occurs more actively in the trabecular bone than in the cortical bone. In the HAp (40%) block, cell infiltration occurred at 4 weeks and even bone formation was observed in the macropores at the center of the samples at 12 weeks; however, new bone appeared to be immature. However, when the CO_3_Ap (0%) block was inserted, new bone was formed only around the samples at 4 weeks. In general, 3D macroporous scaffolds provide greater advantages in the repair of bone defects because the interconnected macroporous structures facilitate nutrient delivery, cell migration, and eventually vascularization and bone ingrowth [45,46,47].

Porous blocks with organized macropores have been fabricated by 3D printing [48,49,50,51]. Currently, this method cannot produce macropores smaller than ~350 μm [48,49,50,51]. In contrast, the method proposed in this study can control the macropore size of porous blocks, within the range of a few micrometers to several hundred micrometers, by using spherical phenol resin with the desired diameter. Wang et al. reported that the trabecular thickness was 0.083 mm when the porous block fabricated by 3D printing was implanted [51]. In this study, when CO_3_Ap (30%) and CO_3_Ap (40%) blocks were implanted, the trabecular thicknesses were 0.15 and 0.17 mm, respectively. These findings demonstrate that CO_3_Ap blocks have a higher osteogenic ability than porous blocks fabricated by 3D printing.

## 5. Conclusions

Macroporous CO_3_Ap blocks were fabricated through a two-step dissolution–precipitation reaction using gypsum as a precursor and spherical phenol resin as a porogen. Material resorption and new bone formation were quicker in the case of CO_3_Ap (30%) and CO_3_Ap (40%) compared to those in CO_3_Ap (0%) and HAp (40%). Treatment of the bone defect was completed within 12 weeks through reconstruction using CO_3_Ap (30%) and CO_3_Ap (40%). Macropores are useful for the acceleration of this process, which is similar to bone remodeling.

## Figures and Tables

**Table 1:** Porosity and diametral tensile strength (DTS) of CO_3_Ap and HAp blocks used in this study. LPS: spherical phenol resin.

LPS (Mass %)	Porosity (%)				DTS (MPa)			
	CaSO_4_	CaCO_3_	CO_3_Ap	HAp	CaSO_4_	CaCO_3_	CO_3_Ap	HAp
0	37.1 ± 2.4	49.0 ± 1.0	48.8 ± 4.5	-	3.4 ± 0.5	2.3 ± 0.4	3.5 ± 0.3	-
30	66.7 ± 1.8	71.4 ± 1.3	67.5 ± 0.7	-	0.8 ± 0.2	0.3 ± 0.1	1.4 ± 0.2	-
40	73.2 ± 0.8	79.1 ± 1.0	75.8 ± 0.9	64.2 ± 1.0	0.5 ± 0.1	0.2 ± 0.1	0.7 ± 0.1	1.8 ± 0.3
